# Importance of the Sequence-Directed DNA Shape for Specific Binding Site Recognition by the Estrogen-Related Receptor

**DOI:** 10.3389/fendo.2017.00140

**Published:** 2017-06-20

**Authors:** Kareem Mohideen-Abdul, Karima Tazibt, Maxime Bourguet, Isabelle Hazemann, Isabelle Lebars, Maria Takacs, Sarah Cianférani, Bruno P. Klaholz, Dino Moras, Isabelle M. L. Billas

**Affiliations:** ^1^Centre for Integrative Biology (CBI), Department of Integrated Structural Biology, Institute of Genetics and of Molecular and Cellular Biology (IGBMC), Illkirch, France; ^2^Centre National de la Recherche Scientifique (CNRS) UMR 7104, Illkirch, France; ^3^Institut National de la Santé et de la Recherche Médicale (INSERM) U964, Illkirch, France; ^4^Université de Strasbourg, Strasbourg, France; ^5^Laboratoire de Spectrométrie de Masse BioOrganique, Centre National de la Recherche Scientifique (CNRS), IPHC UMR 7178, Strasbourg, France

**Keywords:** nuclear receptors, estrogen-related receptor, homodimerization, steroid receptors, DNA recognition, DNA shape, minor groove shape recognition

## Abstract

Most nuclear receptors (NRs) bind DNA as dimers, either as hetero- or as homodimers on DNA sequences organized as two half-sites with specific orientation and spacing. The dimerization of NRs on their cognate response elements (REs) involves specific protein–DNA and protein–protein interactions. The estrogen-related receptor (ERR) belongs to the steroid hormone nuclear receptor (SHR) family and shares strong similarity in its DNA-binding domain (DBD) with that of the estrogen receptor (ER). *In vitro*, ERR binds with high affinity inverted repeat REs with a 3-bps spacing (IR3), but *in vivo*, it preferentially binds to single half-site REs extended at the 5′-end by 3 bp [estrogen-related response element (ERREs)], thus explaining why ERR was often inferred as a purely monomeric receptor. Since its C-terminal ligand-binding domain is known to homodimerize with a strong dimer interface, we investigated the binding behavior of the isolated DBDs to different REs using electrophoretic migration, multi-angle static laser light scattering (MALLS), non-denaturing mass spectrometry, and nuclear magnetic resonance. In contrast to ER DBD, ERR DBD binds as a monomer to EREs (IR3), such as the *tff1* ERE-IR3, but we identified a DNA sequence composed of an extended half-site embedded within an IR3 element (embedded ERRE/IR3), where stable dimer binding is observed. Using a series of chimera and mutant DNA sequences of ERREs and IR3 REs, we have found the key determinants for the binding of ERR DBD as a dimer. Our results suggest that the sequence-directed DNA shape is more important than the exact nucleotide sequence for the binding of ERR DBD to DNA as a dimer. Our work underlines the importance of the shape-driven DNA readout mechanisms based on minor groove recognition and electrostatic potential. These conclusions may apply not only to ERR but also to other members of the SHR family, such as androgen or glucocorticoid, for which a strong well-conserved half-site is followed by a weaker one with degenerated sequence.

## Introduction

The binding of DNA by nuclear receptors (NRs) is essential for the proper transcriptional regulation of the expression of target genes involved in crucial physiological and metabolic pathways (1–3). Specific DNA recognition encompasses several levels of complexity and involves at first place a direct readout mechanism, where the NRs bind specific genomic sequences, known as response elements (REs) and make sequence-specific contacts with the major groove by the formation of base- and amino acid-specific interactions (4–7). Most NRs bind DNA on their cognate RE, that are composed of two hexanucleotide half-sites, either as homodimers or as heterodimers with the ubiquitous partner RXR. Cooperative dimerization of the NRs on DNA increases the repertoire of binding sites that can be bound in a specific manner and leads to more efficient DNA binding. However, a few NRs can also bind DNA efficiently as monomers (7, 8). In this case, the receptors make use of an additional region of interaction between the C-terminal extension (CTE) of the NR and the 3-bp extension at the 5′-end of the RE. The estrogen-related receptors (ERRs) are orphan NRs that are structurally related to the classical estrogen receptors (ERs) and have been categorized as monomeric receptors (9–11). In fact, the ERRs have a marked preference for the recognition of the sequence TNAAGGTCA, which is referred as the estrogen-related response element (ERRE) and corresponds to the classical 6-bp half-site AGGTCA and a 3-bp 5′-extension TNA, where *N* = C is the preferred nucleotide (12–14). The ERRs can also bind *in vitro* with high affinity to the inverted repeat 3 (IR3) REs, which are bound by the ERs and are composed of two hexanucleotide half-sites of consensus sequence AGGTCA organized as inverted repeats separated by a 3-bp spacer (15, 16). However, *in vivo*, a very small overlap exists between the set of genes bound by ERRs and ERs, and when it does, direct overlap occurs through an ERRE embedded within an ERE, leading to fine tuning of the expression of a small set of genes (12). The ERRs do bind the genome strongly and in a widespread and ligand-independent manner. The genes controlled by the ERRs are in a large part associated with metabolic and physiological pathways, such as glucose metabolism, mitochondrial activity, and energy sensing. Therefore, the ERRs represent major transcriptional regulators of energy metabolism in response to physiological and/or environmental challenges (17–23).

At the structural level, the understanding of the ERR function has relied on studies of the isolated DNA (DBD) and ligand-binding domains (LBD). A single ERRβ DBD structure was solved and showed a monomer bound to a short 13-bp DNA sequence encompassing the 9-bp ERRE (24). On the other hand, the ERR LBD was crystallized as a homodimer (25–29), suggesting that the LBD mediates the main dimerization properties of ERRs. Functional data on full ERRs suggested that the homodimer, and not the monomer, is functionally relevant and capable of interaction with co-regulators, in particular with the coactivator PGC-1. This apparent contradiction between the homodimerization of the full receptor and the identification of monomeric ERR-binding sites represents a puzzling piece of information that has not been elucidated until now. Since the full ERR assembly on DNA engage the DBD which is in principle able to perform DNA recognition, we decided to focus on the isolated DBD bound to different types of REs. Solution studies of ERR DBD by nuclear magnetic resonance (NMR) showed that the isolated DBD is a monomer in the absence of DNA or in the presence of a short sequence encompassing the 9-bp extended half-site (24, 30). This is also the case with the ER which is a monomer in solution in the absence of DNA, but that dimerizes on a palindromic ERE/IR3 (31). Homodimerization of ERs and of the other steroid receptors [the androgen (AR), glucocorticoid (GR), mineralocorticoid (MR), and progesterone (PR) receptors, so called the oxosteroid receptors] on IR3 is strongly cooperative in the presence of a correctly configured DNA site (4, 5, 32–35). Therefore, we wondered whether ERR DBD could homodimerize on certain DNA-binding sites and explored a large panel of DNA sequences onto which ERRα DBD can bind. Here, we report the results of these studies and we show, by using complementary biophysical and structural techniques, that stabilization of a dimer of ERRα DBD is possible on specific sequences. Several natural sequences of ERR REs have been considered in our analysis, and to gain deeper insight into the key determinants for ERR DBD dimer formation on DNA, we further designed mutant and chimera DNA sequences that helped us in delineating the critical region for the stabilization of the DBD dimer. In particular, we uncovered that the sequence-driven shape of DNA at the level of the site that is potentially bound by the second DBD subunit is essential for such a process. Thus, ERRα DBD uses information in the minor groove to achieve DNA-binding and concomitant dimer stabilization. Since the natural DNA-binding sites of the NRs are often degenerated with respect to the consensus sequence, somehow accounting for their specific transcriptional output, our results provide a nice example of DNA shape recognition and offer perspectives in the understanding of NR DNA recognition.

## Materials and Methods

### Cloning, Protein Expression, and Purification

Mouse mammary ERRα DBD (70–170) was cloned in the in-house expression vector pnEAtH. The vector was transformed in *Escherichia coli* BL21 (DE3) pRARE2 strain, grown at 37°C and induced for protein expression at OD_600nm_ = 0.6 with 1 mM IPTG at 25°C for 3 h. The cell pellet was resuspended in binding buffer (20 mM Tris pH = 8.0, 400 mM NaCl, 10% glycerol, 4 mM CHAPS) and lysed by sonication. The crude extract was centrifuged at 45,000 × *g* for 1 h at 4°C. The lysate was loaded on a Ni affinity step on HisTrap FF crude column (GE Healthcare, Inc.), and the protein was eluted at a concentration of 150 mM imidazole. The hexahistidine tag was cleaved overnight using thrombin protease. ERRα-DBD was subjected to a Heparin column using an increasing salt gradient. Finally, the ERRα-DBD was polished by size-exclusion chromatography (SEC) in a SEC buffer (50 mM Bis Tris pH = 7.0, 120 mM KCl, 0.5 mM CHAPS, 4 mM MgCl_2_) by using a Superdex S75 16/60 column (GE Healthcare). Complexes were formed by mixing the appropriate DNA solution to the purified ERRα DBD to achieve desired DNA:protein molar ratios. The complex was incubated at least 30 min at 4°C before performing the respective experiments.

### DNA Annealing

HPLC purified single-stranded DNAs were ordered from Sigma-Aldrich, Inc. or Euromedex France. The single-stranded DNA oligonucleotide and its respective anti-sense DNA fragment were mixed in the DNA annealing buffer (10 mM Tris pH = 8.0, 100 mM NaCl, 1% DMSO, 0.1 mM EDTA), heated at 95°C, and gradually cooled down to 4°C using a BIO-RAD PCR machine. 4 mM MgCl_2_ was added to the cooled annealed DNA sample.

### Polyacrylamide Native Gel Electrophoresis

The protein–DNA complexes were run on an 8% polyacrylamide gel (PAGE) at 2 W constant power after pre-running the gel for 40 min at 4°C. Two different native gel systems were used (i) Tris/CAPS (pH = 9.4) buffer system contained 60 mM Tris base and 40 mM CAPS (3-cyclohexil-amino-1-propane-sulfonic acid) (ii) Ammonia/CAPS buffer (pH = 10.4) system (containing 37 mM ammonia and 60 mM CAPS). Approximately 3–5 µg protein was loaded per lane along with its DNA counterpart at defined molar ratios. The polyacrylamide gels were stained using Instant Blue Protein Stain (Expedeon Protein Solutions) for 15 min and rinsed in water.

### SEC Coupled to Multi-Angle Laser Light Scattering

Size-exclusion chromatography (SEC)-MALLS/QELS experiments were performed on a multi-angle laser light scattering detector (miniDAWN TREOS, Wyatt Technologies) coupled in-line with SEC and an interferometric refractometer (Optilab T-rEX, Wyatt Technologies). A Superdex S75 or S200 10/300 GL column (total volume 24 mL, GE Healthcare) with a flow rate of 0.5 mL/min was used to separate the sample before performing the MALLS/QELS measurement. Experiments were done with 50–100 µL receptor–DNA complex samples at concentrations between 1 and 3 mg/mL in 20 mM Bis Tris pH = 7.0, 120 mM KCl, 1 mM MgCl_2_, 0.5 mM CHAPS, 1 mM TCEP. The molar mass was determined by construction of Debye plot using Zimm formalism [plot of K*c/R(θ) as a function of sin^2^(θ/2)] at 1-s data interval. The analysis of the data was performed using the ASTRA 6.1software (Wyatt Technologies).

### Native Electrospray Ionization Mass Spectrometry

Native nano-Electrospray-Mass Spectrometry (nanoESI-MS) analyses were performed on an electrospray quadrupole-time-of-flight mass spectrometer (Synapt G2 HDMS, Waters, Manchester, UK) coupled to an automated chip-based nanoESI source (Triversa Nanomate, Advion, Ithaca, NY, USA). The mass spectrometer was calibrated using singly charged ions produced by a 2-g/L solution of cesium iodide (Acros organics, Thermo Fisher Scientific, Waltham, MA, USA) in 2-propanol/water (50/50 v/v). Samples were buffer exchanged in 150 mM ammonium acetate (NH_4_Ac), pH 7.1 buffer using 0.5 mL Zeba™ Spin desalting Columns (Thermo Fisher Scientific, Waltham, MA, USA) and centrifuged at 1,500 × *g* for 2 min. After buffer exchange, concentrations were determined by UV-Vis using a Nanodrop 2000 Spectrophotometer (Thermo Fisher Scientific, Waltham, MA, USA). Samples were diluted to 5 µM in 150 mM ammonium acetate, pH = 7.1 buffer and directly infused into the mass spectrometer. Instrumental parameters were optimized for the detection of labile non-covalent complexes by raising the interface pressure to 6 mbar and the cone voltage to 140 V. Data treatment was realized with MassLynx 4.1 software (Waters, Manhester, UK).

### Nuclear Magnetic Resonance

Nuclear magnetic resonance experiments were recorded at 700 MHz on an Avance III Bruker spectrometer equipped with a TCI z-gradient cryoprobe. NMR data were processed using TopSpin (Bruker) and analyzed with Sparky software packages (36). NMR experiments were performed in 50 mM sodium phosphate buffer (pH 7.0), 5 mM MgCl_2_, and 1 mM TCEP in 90/10 H_2_O/D_2_O. PAGE-purified DNAs were annealed by heating at 95°C and snap-cooled at 4°C. The concentration of DNA samples was measured using a Nanodrop Spectrometer and calculated with molar extinction coefficients. DNA samples volumes were 150 µL in 3-mm NMR tubes. ^1^H assignments were obtained using standard homonuclear experiments. NMR data were acquired at 15°C and 20°C. Solvent suppression was achieved using the “Jump and Return” sequence combined to WATERGATE (37–39).

Two-dimensional NOESY spectra were acquired with mixing time of 400 and 50 ms. Base pairing was established *via* sequential nuclear Overhauser effects (NOEs) observed in 2D NOESY spectra at different mixing times. Complexes were formed by stepwise addition of ERRα DBD to 100 µM DNA samples, to achieve DNA:protein ratios of 0.5, 1.0, 1.5, and 2.0. The concentrations of protein samples ranged from 0.8 to 1.4 mM maintaining minimal dilution of the DNA samples. All titrations experiments were performed at 20°C, monitoring the imino protons region of 1D spectra.

## Results

### Polyacrylamide Native Gels Suggest the Existence of Different ERR–DNA Complexes Depending on the Nature of the DNA Sequence

**Figure 1:**
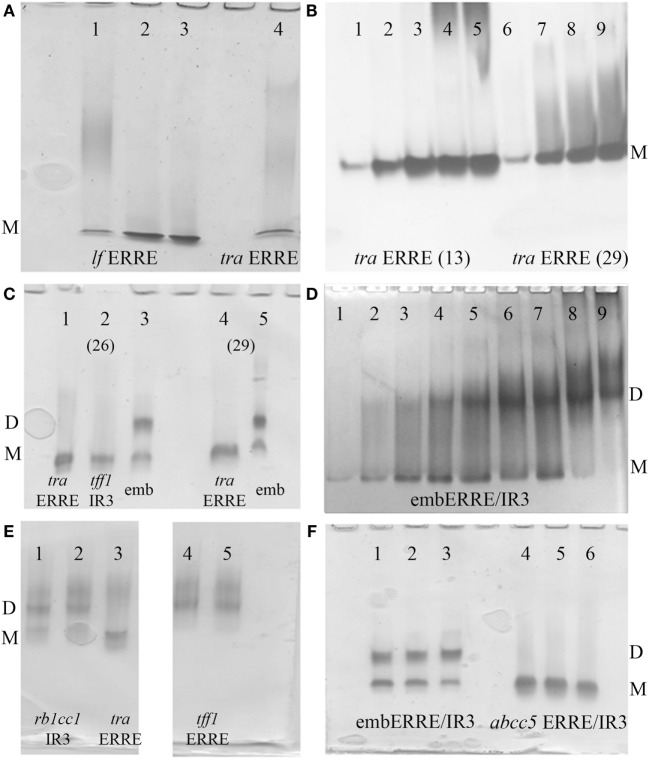
Polyacrylamide native gels. **(A)** Ammonia/CAPS polyacrylamide gel of ERRα DBD-*lf* estrogen-related response element (ERRE) (lanes 1–3) for different DNA:protein ratios 1:1, 0.5:1, and 0.25:1 compared to ERRα DBD-*tra* ERRE DNA:protein ratio = 0.25:1 (lane 4), showing the existence of the monomer only on DNA. **(B)** Effect of DNA length on complex formation for ERRα DBD-*tra* ERRE with 13 bps (lane 1–5) and 29 bps (lane 6–9) for different molar ratios of DNA: DNA-binding domain (DBD), 1:0.5 (lanes 1, 6), 1:1 (lanes 2, 7), 1:2 (lanes 3, 8), 1:3 (lanes 4, 9), and 1:4 (lane 5). **(C)** ERRα DBD complexes were formed with 26-bp DNA fragments of *tra* ERRE (lane 1), *tff1* ERE/IR3 (lane 2), and embERRE/IR3 (lane 3) and with 29-bp fragments of *tra* ERRE (lane 4) and embERRE/IR3 (lane 5) with a molar ratio of DNA: DBD of 1:1. **(D)** ERRα DBD-embedded ERRE/IR3 complexes. Different ratios of DNA:protein were considered for complex formation with the 26 bp fragment originating from the embERRE/IR3 response elements (lanes 1–9: increasing DNA:protein ratios 1:0.25, 1:0.5, 1:1, 1:1.5, 1:2, 1:2.5, 1:3, 1:3.5, and 1:4); showing the appearance of the dimer on DNA. **(E)** Dimer formation is not restricted to the DNA sequence embERRE/IR3, as shown for *rb1cc1* IR3 (lanes 1,2, ratios 1:1 and 0.5:1, respectively) and *tff1* ERRE (lanes 4,5, ratios 1:1 and 0.5:1, respectively) as compared to the strict monomer observed on *tra* ERRE (lane 3, ratio 1:1). **(F)** Comparison of complexes formed between ERRα DBD and embedded ERRE/IR3 DNA sequences, such as embERRE/IR3 (lanes 1–3) and *abcc5* ERRE/IR3 (lanes 4–6) for three different ratios of DNA: DBD (1:1, 0.5:1, and 0.25:1). In all panels, M and D stand for monomer and dimer, respectively.

Estrogen-related receptor binds to IR3 REs encompassing two 6 bps DNA-binding sites separated by 3 bps, as well as to 9 bps extended half-site REs composed of a single specific DNA-binding site of the type TNAAGGTCA (ERRE), the latter REs representing most of the natural REs found in target gene promoters. Here, we first address the issue of complex formation of ERRα DBD-DNA for an extended range of DNA targets, using polyacrylamide Ammonia/CAPS or Tris/CAPS native gel electrophoresis. We considered 33 bp DNA fragments originating from the *tralpha (tra)* and *lactoferrin (lf)* (40, 41) gene promoters (Table S1 in Supplementary Material) as representatives of specific natural ERREs (Figure 1A) and reconstituted complexes between ERRα DBD and DNA using increasing ratios of protein to DNA. For these two different natural ERREs, the ERRα DBD–ERRE complexes migrate as a single band. In addition, we considered two *tra* ERRE fragments of different length, a short one of 13 bp only that solely encompasses the extended 5′-TGAAGGTCA-3′ motif, where only one DBD subunit is allowed to bind and a longer one with 29 bps, which would potentially allow the binding of a DBD dimer (Figure 1B). We observed a single migration band for the 13- and 29-bp ERRE complexes, irrespective of the ratio between DNA and the DBD, from sub-stoichiometric (1:0.5) to higher ratios (up to 1:4). This suggests that a monomer is bound to the 13-bp DNA fragment and remains monomeric in the case of longer DNA fragments (as shown with 29 and 33 bps). No potential dimer formation seems to occur when the amount of DBD subunits is increased. When comparing the migration of ERRα DBD on the ERE/IR3 of the *tff1* gene promoter (16, 42) (*tff1* ERE-IR3) with that of ERRα DBD-*tra* ERRE, a similar migration pattern is observed, suggesting a monomer on this IR3 RE (Figure 1C, lanes 1, 2). In contrast, when considering a composite element made of an extended half-site ERRE embedded into an IR3 RE (embERRE/IR3), we observed a delayed migration of the complex into the gel (Figure 1C, lanes 3, 5). Similarly, when the ratio between the embERRE/IR3 (26 bps here) and DBD is increased, from stoichiometric (DNA:protein = 1:0.25) to higher ratios (up to 1:4), the low migration band disappears and a higher migration band is observed (Figure 1D). These data suggest that a different type of ERRα DBD–DNA complex can be formed on the embERRE/IR3 sequence, as compared to the monomeric ERRα DBD–*tra* ERRE, –*lf* ERRE or –*tff1* ERE-IR3 complexes and likely corresponds to dimeric ERRα DBD on DNA. To gain insight into the unexpected behavior of ERRα DBD on the embERRE/IR3sequence, we explored more sequences belonging to either the IR3-type of REs or to the natural extended ERREs. We found diversity in the migration pattern inside each of the two different types, as shown for *tff1* ERE-IR3 (16, 42) and the *rb1cc1* IR3 (43) as examples of IR3-binding sites, or *tra* ERRE and *tff1* ERRE as examples of ERRE natural DNA REs (Figures 1C,E). While the *tff1* ERE-IR3 and the *tra* ERRE result into a single low migration band, the *rb1cc1* IR3 and the *tff1* ERRE give a more complex migration pattern with mainly two bands, a lower migration band and a delayed migration band similar to what is observed in the case of ERRα DBD-embERRE/IR3. On the other hand, different embedded ERRE/IR3 REs do not necessarily lead to the observation of two migrating species, as shown in Figure 1F, for the natural ERRE/IR3 RE of the *abcc5* gene promoter. Altogether, these data suggest that the existence of a single monomeric species or the occurrence of higher molecular complexes, possibly dimer, on DNA strongly depends on the exact nature of the DNA sequence. In fact, the DNA sequence is likely to be the key factor for the stabilization of ERRα DBD dimer on DNA.

### Electrospray Ionization Mass Spectrometric Analysis Shows the Existence of ERRα DBD Dimer on the Embedded ERRE/IR3

**Figure 2:**
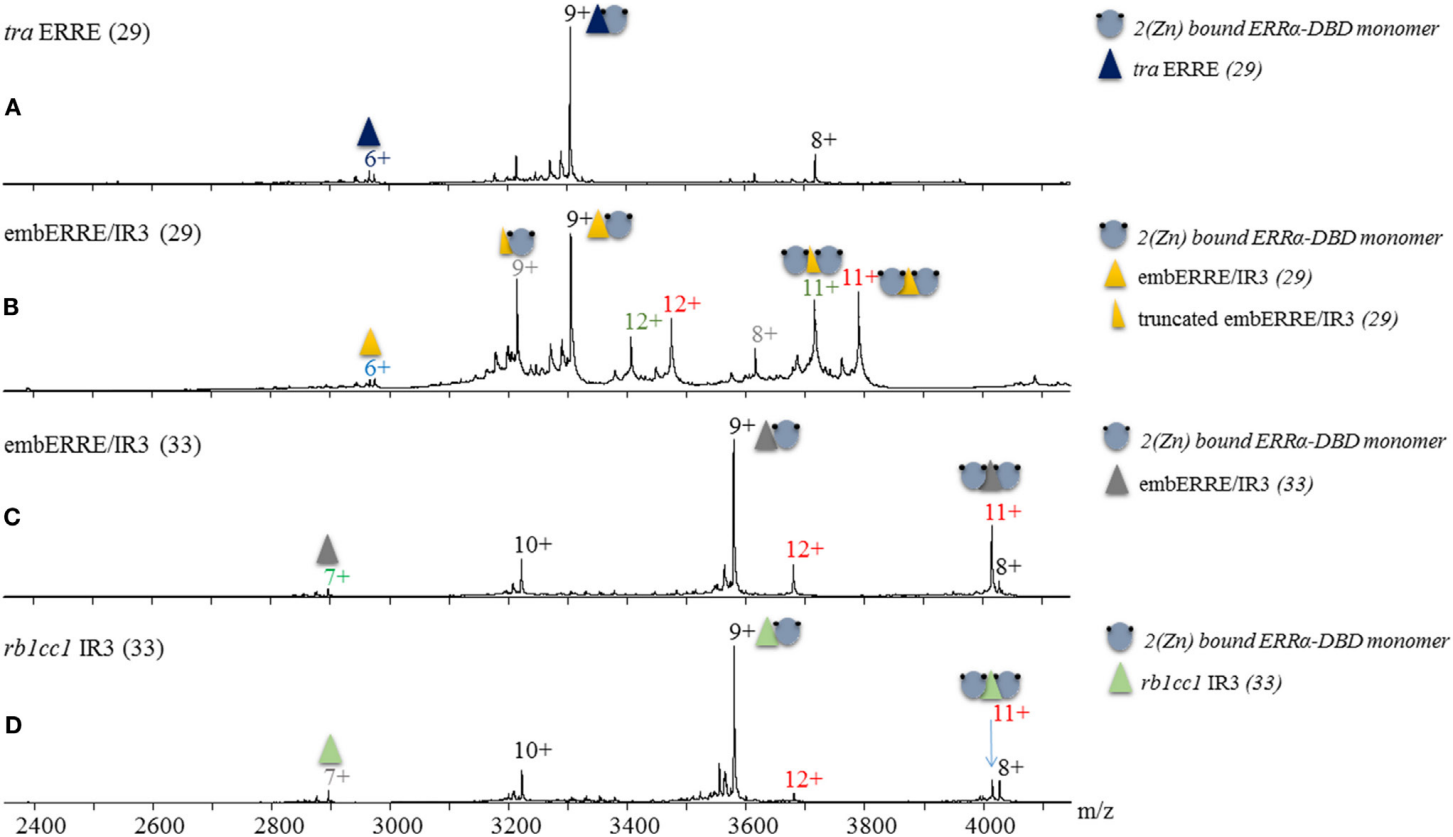
Non-denaturing Mass Spectrometry analysis of ERRα DNA-binding domain (DBD)–DNA complexes. Electrospray ionization mass spectra obtained in non-denaturing conditions of **(A)** ERRα DBD-*tra* estrogen-related response element (ERRE), **(B)** ERRα DBD-embERRE/IR3 (29), **(C)** ERRα DBD-embERRE/IR3 (33), **(D)** ERRα DBD-*rb1cc1* IR3 complexes. The different charged states of the monomeric and dimeric ERRα DBD–DNA complexes are given in black and red, respectively, above the *m/z* peaks. In panel **(B)**, two more species are observed corresponding to DBD monomer (charged state in gray) and dimer (charged state in green) on a truncated DNA lacking one base pair. In all spectra, a small fraction of free DNA is observed (at *m/z* of around 2,900).

Based on our results obtained from native polyacrylamide gels, we wish to assess the exact binding stoichiometry of ERRα DBD on different DNA sequences and first relied on electrospray mass spectrometry (ESI-MS) analysis carried out under non-denaturing conditions. The mass measurement (29,737 ± 1 Da) indicates that the complex between ERRα DBD and *tra* ERRE is composed of a single DBD subunit on the DNA sequence (Figure 2A; Table S2 in Supplementary Material). When ERRα DBD–embERRE/IR3 complexes are considered, MS analysis indicate not only the presence of a monomer but also of a dimer of ERRα DBD on this RE whatever the length of the latter sequence (29 and 33 bps in Figures 2B,C), see Table S1 in Supplementary Material for the sequences and Table S2 in Supplementary Material for the measured masses. The results of the MS analysis are consistent with the biochemical data from the native gel electrophoresis and allow the attribution of the two migration bands observed in the native gels for these complexes as the monomeric and the dimeric ERRα DBD-DNA species. MS analysis further suggests that ERRα DBD can also form a dimer on the IR3 RE of the *rb1cc1* gene promoter (Figure 2D), suggesting that the presence of a dimer is not limited to the embedded RE embERRE/IR3, an observation fully confirmed by the other techniques used in this study.

### SEC Coupled to Multi-Angle Laser Light Scattering Suggests an Equilibrium between Dimer and Monomer Species for Some ERRα DBD–DNA Complexes

**Figure 3:**
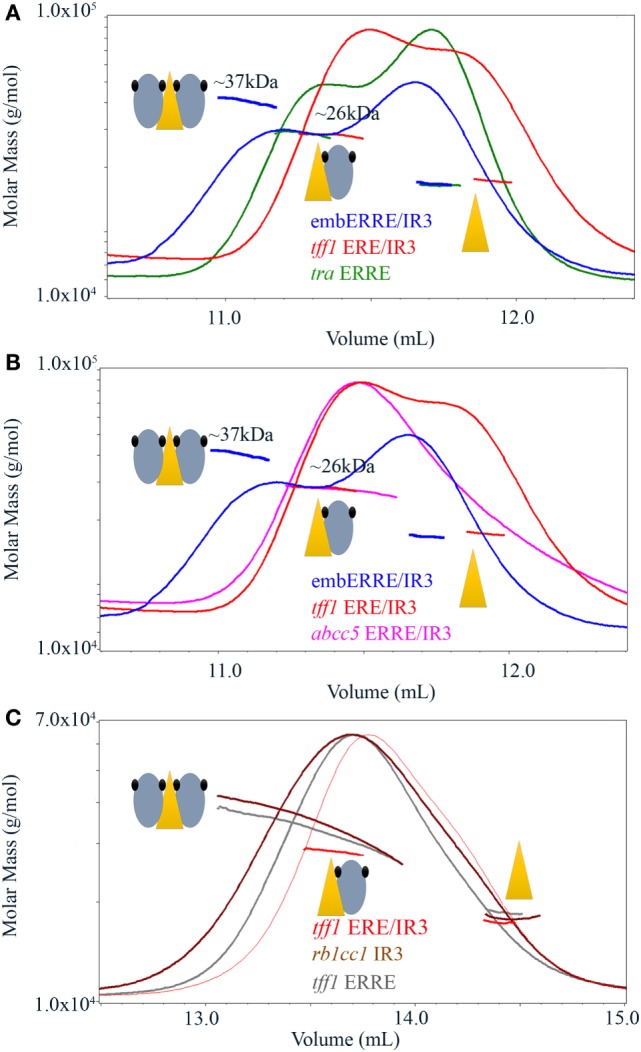
Size-exclusion chromatography-coupled multi-angle laser light scattering of ERRα DNA-binding domain (DBD)–DNA complexes. **(A)** Size-exclusion chromatography (SEC)-MALLS analysis of ERRα DBD bound to embERRE/IR3(26), *tff1* ERE/IR3(26), and *tra* estrogen-related response element (ERRE) (26) (160 µM) showing the elution profile on a SEC S75 10/300 with the direct molar mass measurement of each elution peak. ERRα DBD elutes as a dimer for embERRE/IR3(26) with a measured molar mass of around 37 kDa and as a monomer for *tra* ERRE(26) and *tff1* ERE/IR3(26) with a measured molar mass of 26 kDa. An excess of the 26 bp DNA fragment is seen at the right-hand side of the elution profile. **(B)** SEC-MALLS analysis of ERRα DBD bound to embERRE/IR3(26), *tff1* ERE/IR3(26), and *abcc5* ERRE/IR3(26) (160 µM) showing the elution profile on a SEC S75 10/300 with the direct molar mass measurement of each elution peak. ERRα DBD elutes as a dimer for embERRE/IR3 with a measured molar mass of around 37 kDa, while the embedded *abcc5* ERRE/IR3 elutes as a monomer like *tff1* ERE/IR3(26) with a measured molar mass of 26 kDa. An excess of the 26 bp DNA fragment is seen at the right-hand side of the elution profile. **(C)** SEC-MALLS analysis of ERRα DBD bound to *tff1* ERE/IR3(26), *rb1cc1*IR3(26), and *tff1* ERRE(26) (100 µM) showing the elution profile on a SEC S200 10/300 with the direct molar mass measurement of each elution peak. In contrast to ERRα DBD-*tff1* ERE/IR3(26) that elutes as a monomer with a measured molar mass of 26 kDa, ERRα DBD bound to *rb1cc1* IR3(26) and *tff1* ERRE(26) shows a molar mass that varies along the elution profile from the mass of the dimer on DNA (left) to that of the monomer on DNA (toward the center of the peak). An excess of the 26 bp DNA fragment is seen at the right-hand side of the elution profile.

Mass spectrometric results suggest the existence of monomeric and dimeric ERRα DBD species on the embedded ERRE/IR3 REs and native gels indicate that the existence of larger, possibly dimeric species is not limited to this specific RE, but to other DNAs, as shown for *rb1cc1* IR3 and *tff1* ERRE. Since mass spectrometric analysis in non-denaturing conditions is performed in ammonium acetate, we decided to undertake multi-angle laser light scattering experiments (MALLS) coupled to SEC in more physiological buffer conditions. SEC-MALLS allows the quantitative in-line measurements of the molar mass of the species in solution that are first separated on a SEC column. We first considered ERRα–DBD complexes with 26-bps DNA fragment of either *tra* ERRE, *tff1* ERE/IR3, or embERRE/IR3 and added a rather large excess of DNA while reconstituting the complexes, resulting in a chromatogram with two overlapping peaks, one belonging to the DBD–DNA complex and the other one, on the right, to the free DNA. As shown in Figure 3A, the two complexes ERRα DBD–*tra* ERRE and ERRα DBD–*tff1* ERE/IR3 are composed of one monomer on DNA, while the measured molar mass of ERRα DBD-embERRE/IR3 corresponds to the mass of the dimer. As observed in the native gels (Figure 1F) and shown in Figure 3B, ERRα DBD is a monomer on the embedded ERRE/IR3 RE of the *abcc5* gene promoter. While the binding stoichiometry of all the complexes was rather unambiguous, we wondered how the complexes between ERRα DBD and *rb1cc1* IR3 or *tff1* ERRE would behave since we saw that their migration pattern on the gels was rather complex. As shown in Figure 3C, these measured molar mass of the two complexes reflects the coexistence of dimer (at the left-hand side of the SEC peak) and monomer (toward the center of the peak) on DNA. This suggests that the dimer is less stable on these DNAs when compared to embERRE/IR3 and that both species are in equilibrium in solution. The results of SEC-MALLS experiments are thus consistent with ESI-MS data, and strongly suggest that the dimerization of the isolated ERRα DBD on DNA is possible, but strongly depends on the DNA sequence of the target binding site.

### Binding Properties of ERRα DBD onto DNAs Investigated by NMR

**Figure 4:**
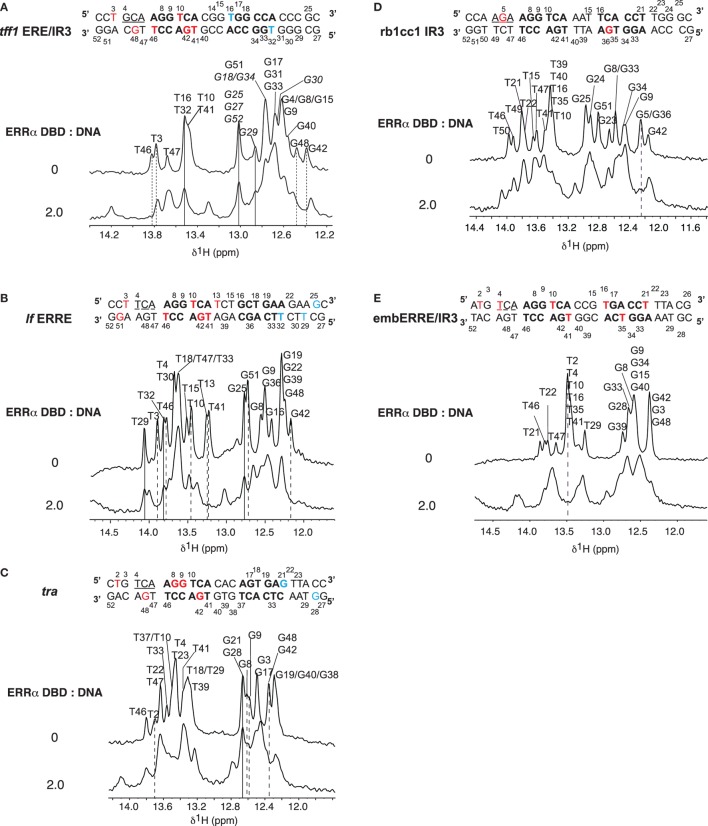
Titrations of DNAs by ERRα DNA-binding domain (DBD). Imino protons region of 1D spectra recorded at 20°C in 50 mM sodium phosphate buffer (pH 7.0), 5 mM MgCl_2_, and 1 mM TCEP in 90/10 H_2_O/D_2_O. The sequences and secondary structures of the DNAs are indicated. The assignments of imino proton based on NOESY experiments are indicated for free DNAs (top). The ratios (ERRα DBD:DNA) are indicated on the left. Broken lines indicate imino protons that undergo chemical shift changes (red in the sequence) and lines indicate imino proton that remain unchanged (blue in the sequence). **(A)** Titration of *tff1* ERE/IR3 (26) DNA. Tentative assignments are indicated in italics. **(B)** Titration of *lf* estrogen-related response element (ERRE) (26) DNA. **(C)** Titration of *tra* ERRE (26) DNA. **(D)** Titration of *rb1cc1* IR3 (26) DNA. **(E)** Titration of embERRE/IR3 (26) DNA.

Nuclear magnetic resonance was used to investigate the binding properties of ERRα DBD onto different types of DNA: *tff1* ERE/IR3, *lf* ERRE, *tra* ERRE, *rb1cc1* IR3, and embERRE/IR3. NMR spectra display imino protons resonances (found between 12 and 14 ppm) provided that they are protected from exchange with the solvent. Their observation indicates that the corresponding protons are involved in hydrogen bonds, generally due to the formation of base pairs. All base paired imino protons of free DNAs were first assigned *via* sequential NOEs observed in 2D NOESY (Figure 4). The A:T Watson–Crick base pair were discriminated from G:C base pair by the strong correlation observable between T H3 imino proton and the H2 proton of adenine. In a G:C Watson–Crick base pair, two strong NOEs are observable between the guanine H1 and the cytosine amino protons. The chemical shifts of imino protons in 1D spectra were then monitored as a function of ERRα DBD concentration and were used to map the DNA regions in contact with the protein. Complexes were formed by stepwise addition of ERRα DBD to DNAs. Successive additions of ERRα DBD on DNAs resulted in several changes in the imino protons region. Addition of ERRα DBD to *tff1* ERE/IR3 DNA induces chemical shifts of the resonances corresponding to T3, T46, G42, and G48 imino protons, indicating that the protein binds the first extended site. In contrast, the resonances corresponding to T16 and T32 imino protons remain unaffected, suggesting that ERRα DBD does not bind the second site (Figure 4A; Figure S1 in Supplementary Material). With *lf* ERRE DNA, most of spectral changes involve nucleotides located in the first extended site. Seven imino protons, T3, T10, T13, T41, G42, T46, and G51 undergo clear chemical shifts, while resonances corresponding to G25, T29, and T32 remain unchanged (Figure 4B; Figure S2 in Supplementary Material). These observations indicate that ERRα DBD binds only the first site. The ERRα DBD titration performed on *tra* ERRE DNA also induces changes in the imino protons region. Despite the complexity of the spectra due to overlapping of several resonances, clear chemical changes are observable for T2, G8, G9, G42, and G48 residues, while G21 and G28 do not undergo variations (Figure 4C; Figure S3 in Supplementary Material). This suggests that ERRα DBD binds the first extended site and not the second. With *rb1cc1* IR3 DNA, similarly to *tra* ERRE DNA, due to overlapping of resonances, we only considered the imino protons that were well resolved. Addition of ERRα DBD to *rb1cc1* IR3 DNA induces a clear chemical shift of the resonance corresponding to G5 and G36 residues (Figure 4D; Figure S4 in Supplementary Material), indicating that the protein contacts the two sites. Finally, titration performed on embERRE/IR3 DNA also induces perturbations in the imino protons region of 1D spectra. Resonances corresponding to T21, T2, T4, T10, T16, T35, and T41 undergo clear chemical shifts upon ERRα DBD binding (Figure 4E; Figure S5 in Supplementary Material). This shows unambiguously that ERRα DBD binds the two sites within the embERRE/IR3 DNA. Taken together, NMR data suggest that the binding of ERRα DBD affects the first extended site in *tff1* ERE/IR3, *lf* ERRE, and *tra* ERRE DNAs, while the protein binds two sites in the case of *rb1cc1* IR3 and embERRE/IR3 DNAs, in perfect agreement with the other biophysical and biochemical data.

### DNA Chimeras to Delineate the Key Molecular Determinants of ERR Dimerization

**Figure 5:**
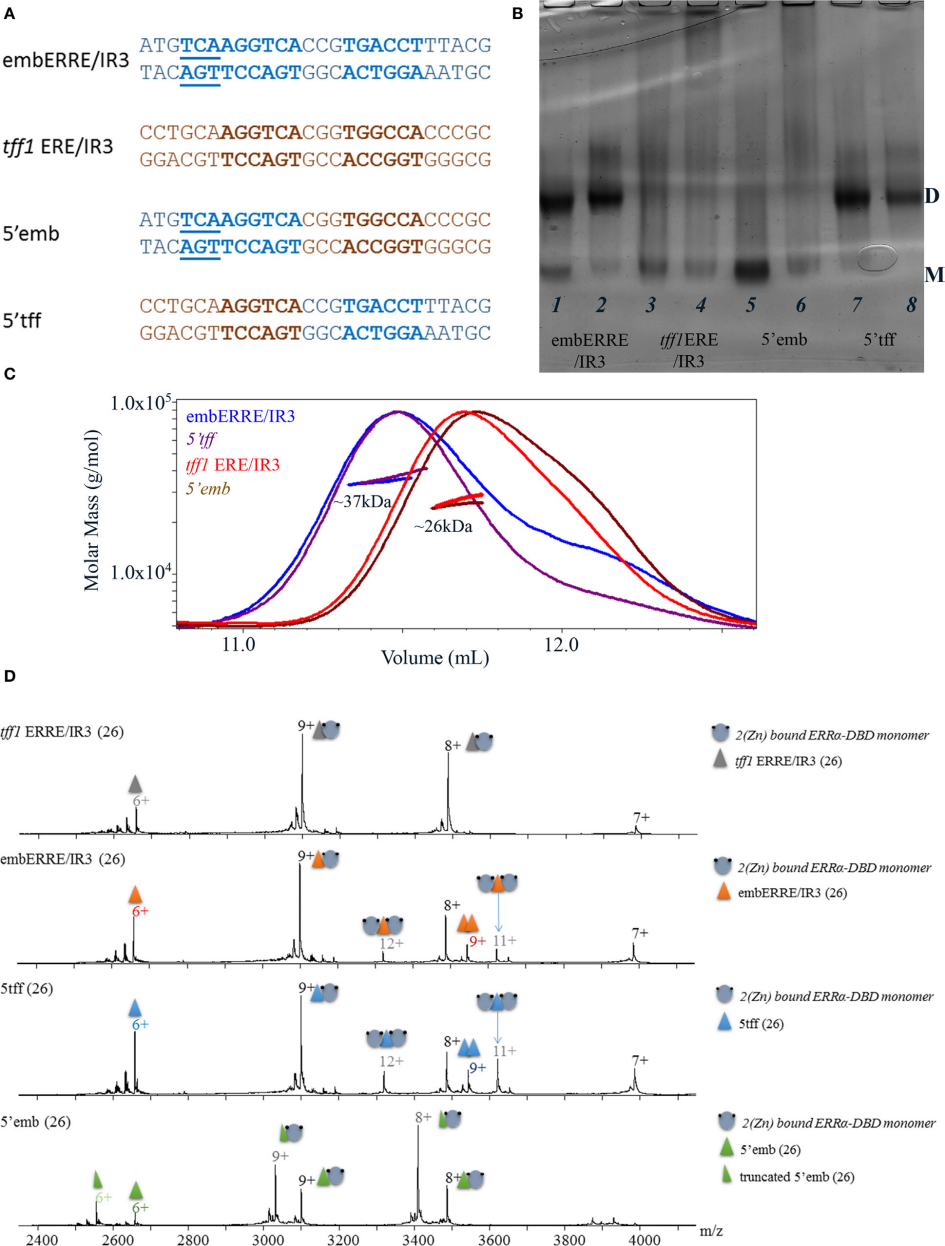
Biochemical and biophysical analysis of DNA chimeras to delineate structural determinant of dimerization. **(A)** Nucleotide sequence of the 26 bp DNA fragments, including the sequence of the embERRE/IR3 (blue), *tff1* ERE/IR3 (brown), 5′emb (5′-end of embERRE/IR3 and spacer and 3′-end of *tff1* ERE/IR3), and 5′tff (5′-end of *tff1* ERE/IR3 and spacer and 3′-end of embERRE/IR3). **(B)** Ammonia/CAPS polyacrylamide gel of the complexes for two different ratios of DNA:protein (1:1 and 0.25:1) for embERRE/IR3 (lanes 1, 2), *tff1* ERE/IR3 (lanes 3, 4), 5′emb (lanes 5, 6), and 5′tff (lanes 7, 8). **(C)** Size-exclusion chromatography (SEC)-MALLS analysis of ERRα DNA-binding domain (DBD) bound to embERRE/IR3 (blue), *tff1* ERE/IR3 (red), 5′tff (magenta), and 5′emb(brown) (160 µM) showing the elution profile on a SEC S75 10/300 with the direct molar mass measurement of each elution peak. ERRα DBD elutes as a dimer for embERRE/IR3 and 5′tff with a measured molar mass of around 37 kDa and as a monomer for *tff1* ERE/IR3 and 5′emb with a measured molar mass of 26 kDa. **(D)** Non-denaturing mass spectrometry analysis of ERRα DBD-DNA complexes for (from top to bottom) *tff1* ERE/IR3, embERRE/IR3, 5′tff, and 5′emb, all being 26-bp long. The different charged states of the monomeric and dimeric ERRα DBD–DNA complexes are given in black and gray, respectively, above the *m/z* peak. In all spectra, a small fraction of free DNA and free DNA dimer is observed (at *m/z* of around 2,900).

For some of the DNA sequences, the ERRα DBD can bind a dimer. This is clearly observed for embERRE/IR3, and to some extend for *tff1* ERRE and *rb1cc1* IR3, but neither for the *tff1* ERE/IR3 nor for *tra* ERRE. Thus, the question arises as to which region is key for the stabilization of a DBD dimer on DNA, since the mere presence of two half-sites is not enough to explain dimerization. Therefore, we considered two DNA sequences, one which is always bound by a monomer (*tff1* ERE/IR3) and another one (embERRE/IR3) which leads to dimer stabilization. We then considered chimeras of these two DNA-binding sequences (Figure 5A), where the 5′ region, including the flanking region and the first half-site belongs to one of the sequence, whereas the 3′ region, including the spacer, the second half-site and the flanking region comes from the other DNA. This results into two chimeras, 5′-*tff1* ERE/IR3_3′embERRE/IR3 (**5′tff**) and 5′embERRE/IR3_3′-*tff1* ERE/IR3 (**5′emb**). The native ammonia/CAPS native gels of the ERRα DBD–DNA complexes indicate that the two chimeras behave differently. The ERRα DBD-5′emb (Figure 5B, lanes 5, 6) behaves as a monomer on DNA, in a way similar to ERRα DBD-*tff1* ERE/IR3 (Figure 5B, lanes 3, 4), while the ERRα DBD-5′tff (Figure 5B, lanes 7, 8) allows the formation of a dimer on DNA, like embERRE/IR3 (Figure 5B, lanes 1, 2). SEC-MALLS experiments confirmed these observations (Figure 5C), where the measured molar masses for the peak are close to the expected values of the monomer or the dimer on DNA. Similarly, the MS analysis performed under non-denaturing conditions confirmed the existence of dimeric species for both ERRα DBD-embERRE/IR3 and ERRα DBD-5′tff complexes (Figure 5D; Table S2 in Supplementary Material). For the latter samples, we noticed that an additional species is present that corresponds to two concatenated DNA molecules. These species come from the initial preparation of DNA (as shown in Figure S6 in Supplementary Material). Taken together, the analysis of the chimeras indicate that the key elements in the DNA sequence for the binding of an ERRα DBD dimer are located in the 3′ region and cannot be attributed to the 5′ 3bp extension present in the embERRE/IR3 sequence.

### The Sequence of the 3′-End Region of DNA Is Crucial for Dimer Stabilization

**Figure 6:**
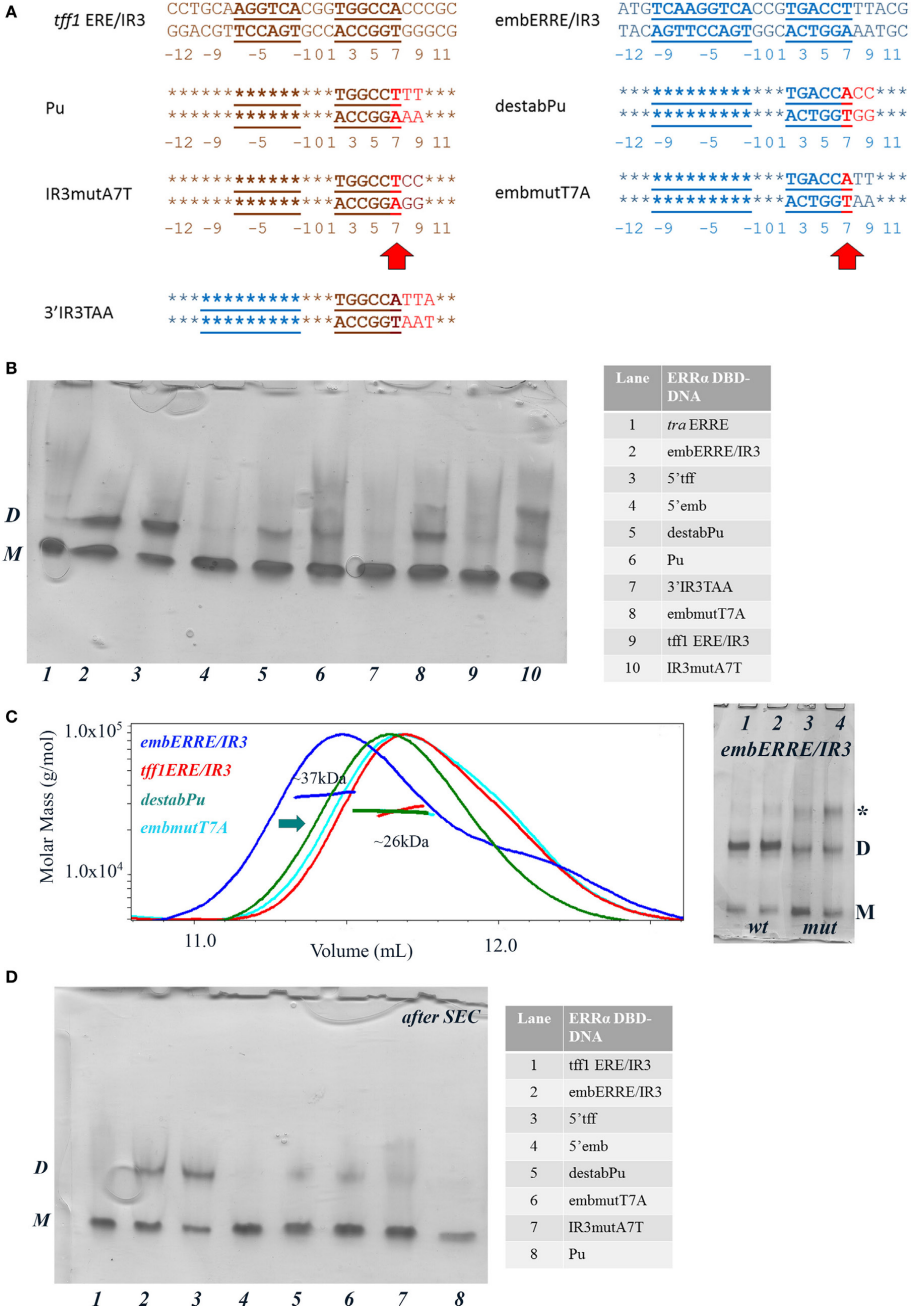
Biochemical and biophysical analysis of DNA mutants suggest that the 3′-end region is crucial for dimer stabilization. **(A)** Nucleotide sequence of the 26-bp DNA mutants, Pu, destabPu, IR3mutA7A, embmutT7A, and 3′IR3TAA. In red are highlighted the changes compared to the parent wild-type sequence. The numbering of the nucleotides is indicated below the DNA sequence. **(B)** Ammonia/CAPS polyacrylamide gel of the complexes for a 1:2 DNA:protein ratio. The complexes, numbered from 1 to 10 are described in the table on the right-hand side. D and M stand for dimer and monomer, respectively. Notice the appearance of aspecific oligomeric species for higher DNA:protein ratios (marked by a star) for some of the DNAs. **(C)** Size-exclusion chromatography (SEC)-MALS analysis of ERRα DNA-binding domain (DBD) bound to embERRE/IR3, *tff1* ERE/IR3, destabPu, and embmutT7A (160 µM) showing the elution profile on a SEC S75 10/300 with the direct molar mass measurement of each elution peak. Mutating embERRE/IR3 at (+7) to (+9), by adding the sequence found in *tff1* ERE/IR3 (in destabPu) or by changing T to A (embmutT7A) leads to the destabilization of the dimeric form of ERRα DBD on DNA (as suggested by the green arrow). On the right-hand side: ammonia/CAPS polyacrylamide gel of ERRα DBD bound to embERRE/IR3 (wt, lanes 1, 2) and embmutT7A (mut, lanes 3, 4) for (1:1) (lanes 1, 3) and (0.5:1) (lanes 2, 4) DNA:protein ratios. D and M stand for dimer and monomer, respectively. Notice the appearance of aspecific oligomeric species for higher DNA:protein ratios (marked by a star). **(D)** Ammonia/CAPS polyacrylamide gel of the complexes after size-exclusion chromatography. The elution peak was fractioned in 40 µL fractions and the fraction corresponding to the maximum of the peak was retained for native gel chromatographic analysis. D and M stand for dimer and monomer, respectively. The complexes, numbered from 1 to 8 are described in the table on the right-hand side.

**Figure 7:**
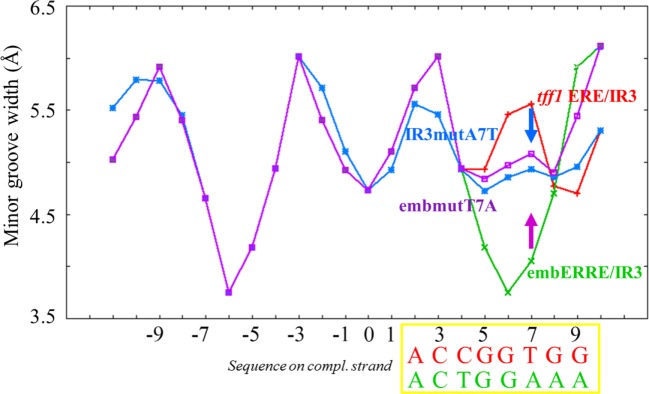
DNA shape predictions of embERRE and *tff1* ERE/IR3 and their respective point DNA mutant embmutT7A and IR3mutA7T. Minor groove width (Å) is plotted as a function of base sequence for embERRE/IR3 (green), *tff1* ERE/IR3 (red), embmutT7A (purple), and IR3mutA7T (blue). The position of the bases is given with respect to the central base pair of the spacer (position 0). The sequences of the complementary strand of *tff1* ERE/IR3 (red) and embERRE/IR3 (green) are given for positions (+2) to (+9) and comprise the second half-site and the two first flanking nucleotides. The purple arrow indicates the destabilization in the dimer formation seen for ERRα DNA-binding domain-embERRE when position (+7) is affected (embmutT7A), while the blue arrow indicates a partial stabilization of dimer formation when *tff1* ERE/IR3 is point-mutated by introducing an T/A at position (+7), resulting to IR3mutA7T.

In order to get a more detailed molecular insight into the key determinants of ERRα DBD dimerization on DNA, we hypothesized that the 3′-region that differs between *tff1* ERE/IR3 and embERRE/IR3 is crucial for dimer binding and designed a series of mutants in this region (Figure 6A). The nucleotides at positions +7 to +10 that include the last base pair of the half-site and the flanking region differ between *tff1* ERE/IR3 and embERRE/IR3. The sequence at these positions is C/G rich with A/T at position +7 for *tff1* ERE/IR3, while for embERRE/IR3 it is composed of three T/A base pairs followed by A/T. This suggests that the complementary strand of embERRE/IR3 between position +5 and +9 contains only purines (+5-GGAAA-+9), while the related sequence of *tff1* ERE/IR3 is G rich with a T in the middle of the stretch at position +7 (+5-GGTGG-+9). Therefore, we first changed the (+5-GGAAA-+9) stretch of embERRE/IR3 to (+5-GGTGG-+9), resulting in the destabPu-embERRE/IR3 (**destabPu**) DNA sequence. In parallel to this, the *tff1* ERE/IR3 GGTGG was modified to GGAAA to mimic locally the sequence seen in embERRE/IR3, leading to *tff1* ERE/IR3-Pu sequence (**Pu**). A significant destabilization of dimer formation is observed for the ERRα DBD–destabPu complex, as seen in the native polyacrylamide gel when comparing the intensity of the dimer band with that of ERRα DBD-embERRE/IR3 (Figure 6B, lanes 2, 5) and measured with SEC-MALLS (Figure 6C), where the SEC peak is shifted to the lower mass range (arrow in Figure 6C). However, the sample fraction collected under the maximum of the SEC peak and run on a native gel indicates that some dimer is present in the sample (lane 5 in Figure 6D). Even more remarkable is the presence of a slight amount of dimer for ERRα DBD-Pu, while the complex with the parent *tff1* ERE/IR3 DNA only shows monomer on DNA (Figure 6B, lanes 6, 9), strongly suggesting that this is the critical region for the stabilization of the second subunit and the dimer formation. The SEC-MALLS of the ERRα DBD-Pu sample shows that the majority of the complexes is composed of monomer on DNA (Figure S7 in Supplementary Material), and no dimer band is seen after the SEC step (Figure 6D, lane 8), suggesting that the stabilization of the dimer is weak on this sequence. We tried to be even more conservative in changing the sequence by mutating only one bp in either *tff1* ERE/IR3 DNA or embERRE/IR3. We chose to mutate the base pair at position (+7) from A/T to T/A for *tff1* ERE/IR3 (**IR3mutA7T**) and from T/A to A/T for embERRE/IR3 (**embmutT7A**) (Figure 6A). The consequences of this mutation are that the complementary DNA stretch between positions (+5) and (+9) becomes GGAGG for IR3mutA7T and GGTAA for embmutT7A. This mutation has already remarkable effects on the dimer association, either slightly destabilizing the dimer for embERRE/IR3 (Figure 6B, lane 8 compared to lane 2 and Figure 6C) or promoting a slight dimer association for *tff1* ERE/IR3 (Figure 6B, lane 10 compared to lane 9). Notice that the effect persists upon SEC-MALLS measurements and sample collection at the peak maximum (Figure 6D, lanes 6, 7). This change in the nature of the nucleotides, either by breaking or restoring the purine stretch observed in the complementary strand between positions (+5) and (+9) is critical. Notice that no dimer is seen to appear, when a TAA extension is present at the 5′end of the second half-site (**3′IR3TAA**) (on the complementary strand and shown in Figure 6B, lane 7). Altogether, the data from native polyacrylamide gels and SEC-MALLS suggest that small variations in the DNA sequence at the 3′-end are linked to the propensity of the DBD to dimerize. Thus, subtle variations in the sequence of this region lead to dramatic effects and support the hypothesis that dimerization of ERRα DBD on DNA is intrinsically linked to the nature of DNA sequence. Since DNA groove dimensions depend on the DNA sequence (44–46), we used a high-throughput, experimentally well validated method to predict the DNA shape features of the sequences considered in our studies (47). As Figure 7 indicates, the analysis shows large differences in the minor groove widths between the *tff1* ERE/IR3 and embERRE/IR3 right in the region between positions (+5) and (+9). The embERRE/IR3 exhibits a pronounced minimum around the nucleotide at position (+6), while the *tff1* ERE/IR3 behaves at contrary at this location with a peak maximum at positions (+6) and (+7). These observations suggest that a narrow minor groove between positions (+6) and (+9) is essential for the stabilization of ERRα DBD homodimer and that wide and shallow minor groove at this location is not favorable to dimer formation. Next, we repeated the DNA shape prediction for the series of mutant and natural sequences. As shown on Figure 7, the mutants embmutT7A and IR3mutA7T follow a quite similar profile with the expected trend, the former exhibiting a shallower minimum as compared to embERRE/IR3 and the latter a strong decrease in the minor groove width as compared to *tff1* ERE/IR3. These predictions are consistent with the observations of the destabilization of the dimeric form for embmutT7A, or on the contrary of stabilization of a fraction of the complex into the dimeric form for IR3mutA7T. Remarkably, the DNA shape predictions for the various individual ERR binding sites considered in our studies are remarkably consistent with the observations of dimer versus monomer-only behavior (see Figure S8 in Supplementary Material).

## Discussion

Previous studies established that the ERRs function as homodimers. In particular, dimerization is required for the transcriptional activity of these receptors (48–51) and for the productive interaction with coactivators (10, 42, 52). The LBD was shown to mediate, in a large part, dimerization and the crystal structures of the ERRs (25, 26, 53–55) demonstrated in a consistent manner that the ERR LBD homodimer has a canonical dimerization interface, similar to the one seen in other NR homo- and heterodimers (56, 57). Concerning the DNA-binding properties, the ERR can bind *in vitro* as a homodimer to palindromic IR3 REs, such as those recognized by the ER (16, 42). However, *in vivo*, the ERR-binding sites are monomeric sites composed of extended half-site sequences (ERRE), and no consensus can be found for a second-binding site (12, 14). In addition, it was suggested that the sequence of the ERRE, and in particular that of the 3-bp extension, dictates the binding preference of ERR as a dimer or as a monomer even though only the dimeric form, and not the monomeric one, was shown to be capable of interacting with the coactivator PGC-1α (10, 42, 51). Thus, the mode of binding of DNA by ERR remained elusive and controversial.

To study the molecular mechanisms of binding and recognition of DNA by ERR, we decided to focus on the DNA-binding mode of the isolated DBD, allowing an unbiased understanding of the intrinsic DNA-binding properties of the protein, independently of the dimerization of the LBD. Using a combined approach of complementary biophysical and structural techniques, we show that ERRα DBD behaves either as a monomer or as a dimer depending on the DNA sequence. This is the first report demonstrating that an ERRα DBD dimer can be formed on DNA and that the dimerization of the DBD is not linked to the mere existence of two hexameric half-sites in the binding sequence onto which the two subunits could reside. We indeed show that on classical IR3 RE, such as the *tff1* ERE/IR3 element, ERRα DBD binds as a monomer, in contrast to what is observed for ER DBD that dimerizes in a strong cooperative manner on this RE (31, 35, 58).

We demonstrate the existence of ERRα DBD homodimer on the embERRE/IR3 binding site which is composed of an extended half-site embedded into an IR3 RE. Binding on this element is observed for different lengths of DNA, indicating that the effect is not biased by a specific oligonucleotide. On the other hand, the fact that the sequence is composed of an extended half-site RE embedded into an IR3 element is not the key requirement that leads to the presence of a homodimer. Importantly, the homodimer can form on different types of REs, i.e., on *rb1cc1* IR3, on *tff1* ERRE, or on embERRE/IR3 and what counts for dimerization, as demonstrated by the studies on the chimera DNAs, is the sequence at the 3′-end where the second ERRα DBD subunit can potentially bind. The analysis of the region of embERRE/IR3, where the second subunit binds, reveals a T/A-rich region of the first three nucleotides of the second half-site on the complementary strand and the two flanking bases (+5-GGAAA-+9). Mutating this region of embERRE/IR3 by introducing an A/T bp at position (+7) leads to the destabilization of the dimeric form (destabPu and embmutT7A). Conversely, mutating the equivalent region of *tff1* ERE/IR3 (+5-GGTGG-+9) to purines only (Pu or IR3mutA7T) leads to a remarkable stabilization of the complex into the dimeric form.

The specific recognition of DNA by proteins involves two mechanisms, one that relies on a specific sequence through base- and amino acid-specific hydrogen bonds and occurs primarily in the major groove and another one that involves sequence-dependent DNA shape effects, like minor groove width variations. Local sequence-dependent minor groove shape variations have indeed been established as a crucial mechanism widely used in the protein–DNA recognition that allows proteins to distinguish small differences in nucleotide sequence (44–46). Further, minor groove width and electrostatic potential are strongly correlated. Narrow minor grooves exhibit an enhanced electronegative potential and form specific binding sites for positively charged amino acids, and in particular for arginine residues. Using the DNA shape method (47) applied to the individual DNA sequences, we demonstrate that the minor groove width is intrinsically correlated to the capacity of the receptor DBD to homodimerize on DNA in an IR3-type of configuration (Figure 7; Figure S8 in Supplementary Material). Crucial nucleotides for dimerization effect are base pairs in the first half of the second binding site and flanking nucleotides. Our data parallel with the recent studies of GR DBD on different natural IR3 REs (GBSs) which report that the flanking nucleotides modulate DNA shape locally by acting on the minor groove width (59), but the extend of the variation in minor groove width seen for GBSs is rather modest as compared to that predicted for the ERR DNA-binding sequences. Interestingly, changing the flanking nucleotides of the GBSs resulted in different relative positioning of the dimer halves with consequences on the dimerization interface. The authors further argued that an intact GR dimerization interface driven by the sequence-dependent DNA shape is crucial for adequate positioning of the two GR subunits and that the functionally relevant positioning of the two subunits differs from the optimal positioning of the two DBDs in the major groove. Here, we show an even more striking effect, because the sequence-dependent DNA shape and the corresponding minor groove width at position (+5) to (+9) is a key factor in the stabilization of the ERRα DBD homodimer on DNA. The structure of the monomeric complex between ERRβ DBD and a short 13-bp consensus recognition sequence of the type ERRE provides the structural bases for the stabilization of a monomer on ERRE, which was shown to strongly depend on additional contacts between residues in the A-box at the CTE, including the Arg–Gly–Gly–Arg motif, and the minor groove at the level of the 3-bp 5′-extension (24). In this article, we show that after the specific binding of the first ERR DBD subunit to the 9 bp ERRE, the dimerization of the second subunit will take place in a cooperative manner on DNA only if the local DNA shape and its intrinsic groove dimensions are adequate. From the analysis of the DNA shape as predicted for individual DNA sequences, we see that dimer formation is favored by a narrow minor groove. Indeed, the embERRE/IR3 sequence between position (+7) and (+9) exhibits an A-tract that favors a narrow minor groove. A-tracts have indeed been identified in a large number of protein–DNA complexes to positively correlate with narrow minor (60) that represent the binding sites of positively charged arginine residues (45, 46). For example, the Hox family of homeodomain-containing transcription factors bind to generic binding sites through major groove-recognition helix interactions, but the specificity in target gene recognition arises from DNA shape and electrostatic potential driven interactions between the N-terminal arm and linker regions of the Hox protein with the minor groove (61), in a way very similar to the one observed here for ERRα DBD and its CTE.

The present studies demonstrate that the different ERR-binding sequences lead to different dimerization properties of the isolated ERRα DBD on DNA. This effect is correlated to the sequence-dependent DNA shape that represents an input signal that dictates the stabilization of the homodimer on DNA. Structure–function studies of GR DBD indicated there is no correlation between *in vitro* affinity and *in vivo* activity of this homodimer on specific DNA sequences (59). This is also the case for ERR, as suggested by *in vitro* binding affinity experiments, which indicate for example that ERR binds with high affinity to the *tff1* ERE/IR3, while exhibiting a weak transcriptional activity on this element (16, 42). However, our studies give further insight into the role of DNA shape which affects the dimerization capability of the isolated DBD.

The consequence for the binding of the full receptor which naturally forms a homodimer through the LBD is that one of the two DBD subunits is anchored in a specific manner to DNA, while the degree of stabilization of the other subunit on DNA depends on the DNA shape that it experiences. Thus, the conformation of the full homodimeric receptor and its dynamic behavior on DNA are likely to be affected. As a result, the interactions with co-regulators could be differentially modulated by providing distinct interactions surfaces. Previous studies on the binding of full ERR to DNA showed that indeed, depending on the sequence of the target elements, ERR exhibits differential affinity for the co-regulators PGC-1 and RIP14, which in turn alter its transcriptional activity (62). This is also the case for other NRs, which show that their binding sequence leads to a differential recruitment of co-regulators at target gene promoters, as e.g., for the ER (63).

**Figure 8:**
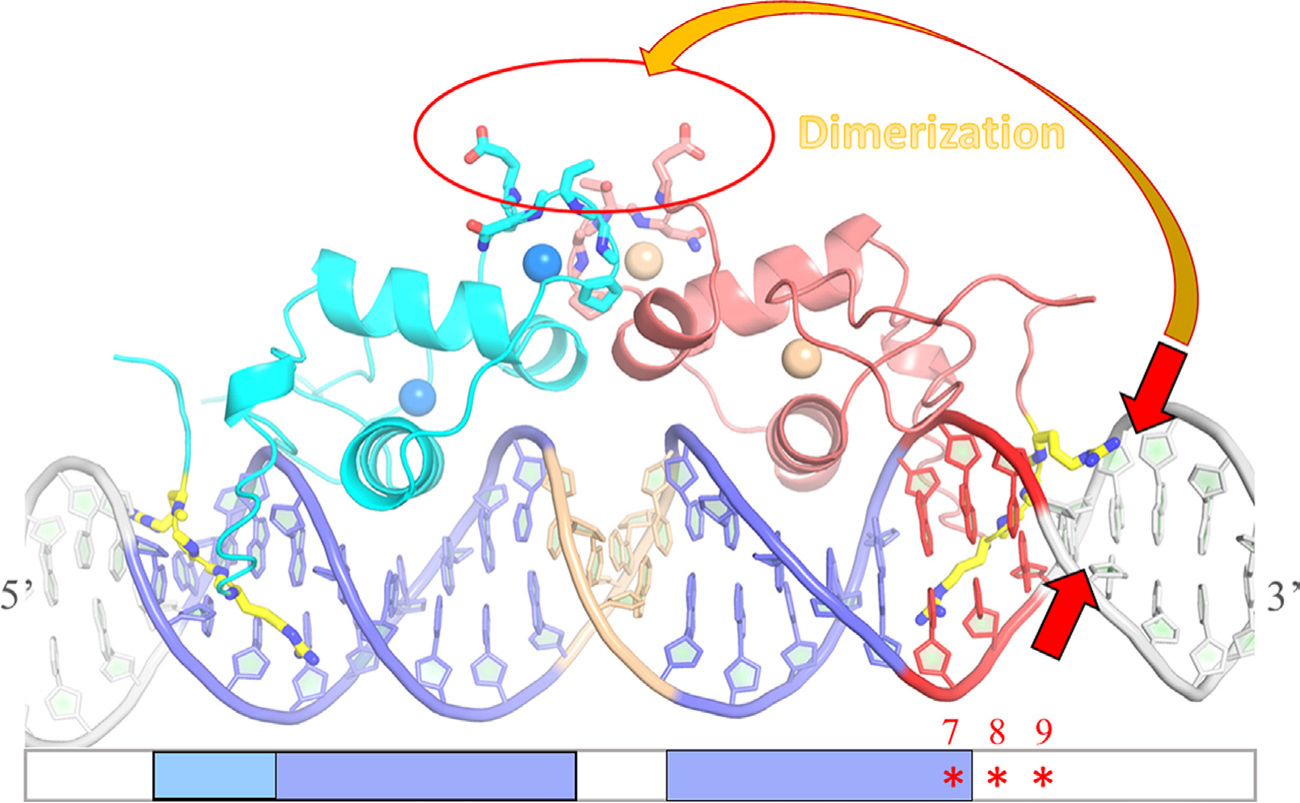
Schematic drawing of the influence of DNA shape on dimer stabilization. Model of the ERRα DNA-binding domain (DBD) dimer on DNA based on the structure of ERα DBD-IR3 response elements (RE) (PDB code 1HCQ), where the nuclear magnetic resonance structure of ERRβ DBD (PDB code 1L01) is superimposed to each of the two subunits of ERα DBD-IR3 RE. An ideal B-DNA model is used in the drawing. Comparative analysis of the residues of the D-box involved in dimerization between ER and ERR suggests that ERRα DBD could *a priori* dimerize in a way similar to that seen in the structure of ERα DBD, without any structural impediment (red circle). On the other hand, the stabilization of ERRα DBD dimer on DNA is influenced by the sequence-dependent DNA shape of the second ERR-binding site at the 3′-end of DNA. The local geometry of the minor groove that encompasses the second half-site and the flanking nucleotides (especially between positions + 5 and + 9) is crucial for the formation of an ERRα DBD dimer on DNA. Sequence-driven narrow minor grooves at this location (small red arrows) favor dimer stabilization (large orange arrow).

In summary, our report demonstrates that the sequence-dependent DNA shape of the ERR-binding site influences the recruitment of ERRα DBD dimer on DNA, by promoting the right conformation of the two subunits on DNA for cooperative interaction and dimer stabilization. The key structural component promoting dimer formation is the local geometry of the minor groove that encompasses the second half-site and the flanking nucleotides. The local minor groove shape strongly depends on the sequence and it is this information imprinted in the DNA shape that ERR uses to achieve DNA-binding specificity (scheme Figure 8). It is tempting to speculate that DNA-binding specificity of other NRs to their target genes is achieved by similar regulatory mechanisms. In particular, the oxosteroid NR family, including the AR, the GR, the MR and the PR receptors bind as homodimer to similar IR3 binding sites that exhibit a well conserved and strong half-site and a second degenerated one that gives oxosteroid receptor specificity (4, 5, 7, 32, 64–66). Our studies on ERR suggest that the local sequence-dependent DNA shape of the second half-site is crucial for receptor specificity in DNA recognition.

## Author Contributions

IB, DM, and BK designed the study and supervised research; IB, KM-A, and DM wrote the manuscript, KM-A, KT, MB, IH, IL, MT, and IB performed the experiments; KM-A, KT, MB, IL, SC, and IB analyzed data.

## Conflict of Interest Statement

The authors declare that the research was conducted in the absence of any commercial or financial relationships that could be construed as a potential conflict of interest.

## Supplementary Material

Figure S1 Titration of *tff1* ERE/IR3 DNA by ERRα DNA-binding domain (DBD). Imino protons region of 1D spectra recorded at 20°C in 50 mM sodium phosphate buffer (pH 7), 5 mM MgCl_2_ and 1 mM TCEP in 90/10 H_2_O/D_2_O. The assignments of imino proton based on NOESY experiments are indicated for free *tff1* ERE/IR3 (top). Tentative assignments are indicated in italics. The ratios (ERRα DBD:*tff1* ERE/IR3) are indicated on the left. Broken lines indicate imino protons that undergo chemical shift changes (red in the sequence) and lines indicate imino proton that remain unchanged (blue in the sequence).

Figure S2 Titration of *lf* ERRE DNA by ERRα DNA-binding domain (DBD). Imino protons region of 1D spectra recorded at 20°C in 50 mM sodium phosphate buffer (pH 7.0), 5 mM MgCl_2_ and 1 mM TCEP in 90/10 H_2_O/D_2_O. The assignments of imino proton based on NOESY experiments are indicated for free *lf* estrogen-related response element (ERRE) (top). The ratios (ERRα DBD: *lf* ERRE) are indicated on the left. Broken lines indicate imino protons that undergo chemical shift changes (red in the sequence) and lines indicate imino proton that remain unchanged (blue in the sequence).

Figure S3 Titration of *tra* estrogen-related response element (ERRE) DNA by ERRα DNA-binding domain (DBD). Imino protons region of 1D spectra recorded at 20°C in 50 mM sodium phosphate buffer (pH 7.0), 5 mM MgCl_2_ and 1 mM TCEP in 90/10 H_2_O/D_2_O. The assignments of imino proton based on NOESY experiments are indicated for free *tra* ERRE (top). The ratios (ERRα DBD: *tra* ERRE) are indicated on the left. Broken lines indicate imino protons that undergo chemical shift changes (red in the sequence) and lines indicate imino proton that remain unchanged (blue in the sequence).

Figure S4 Titration of *rb1cc1* IR3 DNA by ERRα DNA-binding domain (DBD). Imino protons region of 1D spectra recorded at 20°C in 50 mM sodium phosphate buffer (pH 7.0), 5 mM MgCl_2_, and 1 mM TCEP in 90/10 H_2_O/D_2_O. The assignments of imino proton based on NOESY experiments are indicated for free rb1cc1/IR3 (top). The ratios (ERRα DBD: *rbcc1* IR3) are indicated on the left. Broken lines indicate imino protons that undergo chemical shift changes (red in the sequence).

Figure S5 Titration of embERRE/IR3 DNA by ERRα DNA-binding domain (DBD). Imino protons region of 1D spectra recorded at 20°C in 50 mM sodium phosphate buffer (pH 7.0), 5 mM MgCl_2_, and 1 mM TCEP in 90/10 H_2_O/D_2_O. The assignments of imino proton based on NOESY experiments are indicated for free embERRE/IR3 (top). The ratios (ERRα DBD: embERRE/IR3) are indicated on the left. Broken lines indicate imino protons that undergo chemical shift changes (red in the sequence).

Figure S6 Non-denaturing Mass Spectrometry analysis of free DNA. Non-denaturing mass spectrometry analysis of free DNA complexes for **(A)** embERRE/IR3, **(B)** 5′tff, and **(C)** 5′emb, all being 26 bps long, recorded in the same non-denaturing conditions as the protein-DNA complexes are measured (Pi = 6 mbar; Vc = 140V). The charged states are indicated above the peaks. A small fraction of DNA dimer is observed under these experimental conditions.

Figure S7 Size-exclusion chromatography-coupled multi-angle laser light scattering of ERRα DNA-binding domain (DBD)-DNA complexes. SEC-MALLS analysis of ERRα DBD bound to 26 bp embERRE/IR3 (blue), *tff1* ERE/IR3 (red), embmutT7A (green), IR3mutA7T (cyan), and Pu (dark blue) (160 µM), showing the elution profile on a SEC S75 10/300 with the direct molar mass measurement of each elution peak. The elution profiles of the complexes with the mutant DNA complexes are eluted slightly earlier as *tff1* ERE/IR3-ERRα DBD and the fraction of complex collected at the peak maximum is seen to contain a small fraction of dimeric DBD on DNA (see Figure 6D).

Figure S8 DNAshape predictions of other DNA fragments. Minor groove width (Å) is plotted as a function of base sequence for **(A)** *rb1cc1* IR3 (blue) and compared to those of embERRE/IR3 (green) and *tff1* ERE/IR3 (red). The position of the bases is given with respect to the central bp of the spacer (position 0). The sequences of the complementary strand of *rb1cc1* IR3 (blue) and embERRE/IR3 (green) are given for positions (+2) to (+9) and comprise the second half-site and the two first flanking nucleotides; **(B)** *tra* estrogen-related response element (ERRE) (blue) and *tff1* ERRE (green) compared to those of embERRE/IR3 (red). The position of the bases is given with respect to the central bp of the spacer (position 0). The sequences of the complementary strand of *tra* ERRE (blue) and *tff1* ERRE (green) are given for positions (+2) to (+9) and comprise the second half-site and the two first flanking nucleotides; **(C)** *abcc5* ERRE/IR3 (blue) and compared to those of embERRE/IR3 (green) and *tff1* ERE/IR3 (red). The position of the bases is given with respect to the central bp of the spacer (position 0). The sequences of the complementary strand of *abcc5* ERRE/IR3 (blue) and embERRE/IR3 (green) are given for positions (+2) to (+9) and comprise the second half-site and the two first flanking nucleotides; **(D)** the mutants destabPu (blue) and Pu (purple) and compared to those of embERRE/IR3 (green) and *tff1* ERE/IR3 (red). The position of the bases is given with respect to the central bp of the spacer (position 0). The sequences of the complementary strand of destabPu (blue) and Pu (purple) are given for positions (+2) to (+9) and comprise the second half-site and the two first flanking nucleotides.
