# Supplementary material for: Importance of the Sequence-Directed DNA Shape for Specific Binding Site Recognition by the Estrogen-Related Receptor
Source: Front Endocrinol (Lausanne). 2017 Jun 20;8:140. doi: 10.3389/fendo.2017.00140 (PMC5476932; doi:10.3389/fendo.2017.00140)
Supplement: Supplementary file 1 [file Table_1.pdf]

Table S1. List of the DNA sequences used in the different studies

DNA name (length)	Sequence	RE type/gene
tra ERRE (13)	GCTCAAGGTCAC<br>CGAGTTCCAGTG	ERRE/TRalpha
tra ERRE (26)	CTGTCAAGGTCACACAGTGAGTTACC<br>GACAGTTCCAGTGTGTCACTCAATGG	ERRE/TRalpha
tra ERRE (29)	AATTGTCAAGGTCACACAGTGAGTTACCG<br>AACAGTTCCAGTGTGTCACTCAATGGCTT	ERRE/TRalpha
tra ERRE (33)	CGATTTCTCAAGGTCACACAGTGAGTTACCGGC<br>GCTAAACAGTTCCAGTGTGTCACTCAATGGCCG	ERRE/TRalpha
lf ERRE (33)	GGCACCTTCAAGGTCATCTGCTGAAGAAGATAG<br>CCGTGGAAGTTCCAGTAGACGACTTCTTCTATC	ERRE/lactoferrin
tff1 ERRE (26)	GGATTAAGGTCAGGTTGGAGGAGACG<br>CCTAATTCCAGTCCAACCTCCTCTGC	ERRE/tff1-ps2
tff1 ERE/IR3 (26)	CCTGCAAGGTCACGGTGGCCACCCGC<br>GGACGTTCCAGTGCCACCGGTGGGCG	IR3/tff1-ps2
rb1cc1 IR3 (26)	CCAAGAAGGTCAAATTCACCTTGGGC<br>GGTTCTTCCAGTTTAAGTGGAACCCG	IR3/rb1cc1
rb1cc1 IR3 (33)	TCCGCAAAGAAGGTCAAATTCACCTTGGGAAAG<br>AGGCGTTTCTTCCAGTTTAAGTGGAACCCTTTC	IR3/rb1cc1
embERRE/IR3 (26)	ATGTCAAGGTCACCGTGACCTTTACG<br>TACAGTTCCAGTGGCACTGGAAATGC	embedded<br>ERRE/IR3
embERRE/IR3 (29)	ATTGTCAAGGTCACCGTGACCTTTACCGA<br>AACAGTTCCAGTGGCACTGGAAATGGCTT	embedded<br>ERRE/IR3

embERRE/IR3 (33)	GAAATTG**TCAAGGTCA**CCG**TGACCT**TTACCGCC<br>CTTTAAC**AGTTCCAGT**GGC**ACTGGA**AATGGCGG	embedded<br>ERRE/IR3
abcc5 ERRE/IR3 (26)	CCG**TGAAGGTCA**TCG**TAGCCC**GCGTC<br>GGC**ACTTCCAGT**AGC**ATCGGG**CGCAG	embedded<br>ERRE/IR3/abcc5
5'tff (26)	CCTGCA**AGGTCA**CCG**TGACCT**TTACG<br>GGACGT**TCCAGT**GGC**ACTGGA**AATGC	Chimera
5'emb (26)	ATG**TCAAGGTCA**CGG**TGGCCA**CCCGC<br>TAC**AGTTCCAGT**GCC**ACCGGT**GGGCG	Chimera
destabPu (26)	ATG**TCAAGGTCA**CCG**TGACCACC**ACG<br>TACAGTTCCAGTGGC**ACTGGT**GGTGC	mutant
Pu (26)	CCTGCA**AGGTCA**CGG**TGGCCT**TTCGC<br>GGACGT**TCCAGT**GCC**ACCGGA**AAGCG	mutant
IR3mutA7T(26)	CCTGCA**AGGTCA**CGG**TGGCCT**CCCGC<br>GGACGT**TCCAGT**GCC**ACCGGA**GGGCG	mutant
embmutT7A(26)	ATG**TCAAGGTCA**CCG**TGACCA**TTACG<br>TAC**AGTTCCAGT**GGC**ACTGGT**AATGC	mutant
3'IR3TAA(26)	ATG**TCAAGGTCA**CGG**TGGCCA**TTAGC<br>TAC**AGTTCCAGT**GCC**ACCGGT**AATCG	mutant
