# Supplementary material for: Importance of the Sequence-Directed DNA Shape for Specific Binding Site Recognition by the Estrogen-Related Receptor
Source: Front Endocrinol (Lausanne). 2017 Jun 20;8:140. doi: 10.3389/fendo.2017.00140 (PMC5476932; doi:10.3389/fendo.2017.00140)
Supplement: Supplementary file 2 [file Table_2.pdf]

Table S2. List of the measured masses by ESI-MS

Samples	Truncated DNA RE (Da)#	DNA RE (Da) #	DNA RE dimer (Da) #	ERRα-DBD - Truncated DNA 1 :1 complex (Da) #	ERRα-DBD + DNA 1 :1 complex (Da) #	ERRα-DBD + DNA 2:1 complex (Da) #
tff1 ERE/IR3	-	15 947	-	-	27 893	-
ERRα-DBD + embERRE/IR3	-	15 941	31 883	-	27 885	39 829
ERRα-DBD + 5ttf'	-	15 943	31 887	-	27 888	39 831
ERRα-DBD + 5'emb	15 327	15 945	-	27 738	27 889	-
ERRα-DBD + *tra* ERRE (29)	-	17 794	-	29 737	-	-
ERRα-DBD embERRE/IR3(29)	16 977	17 794	28 921	29 738	40 866	41 686
ERRα-DBD embERRE/IR3(33)	-	20 268	-	32 212	-	44 157
ERRα-DBD + *rb1cc1* IR3 (33)	-	20 265	-	32 209	-	44 153

#Mass uncertainty ± 1 Da
