# Supplementary figures and images for: Importance of the Sequence-Directed DNA Shape for Specific Binding Site Recognition by the Estrogen-Related Receptor
Source: Front Endocrinol (Lausanne). 2017 Jun 20;8:140. doi: 10.3389/fendo.2017.00140 (PMC5476932; doi:10.3389/fendo.2017.00140)

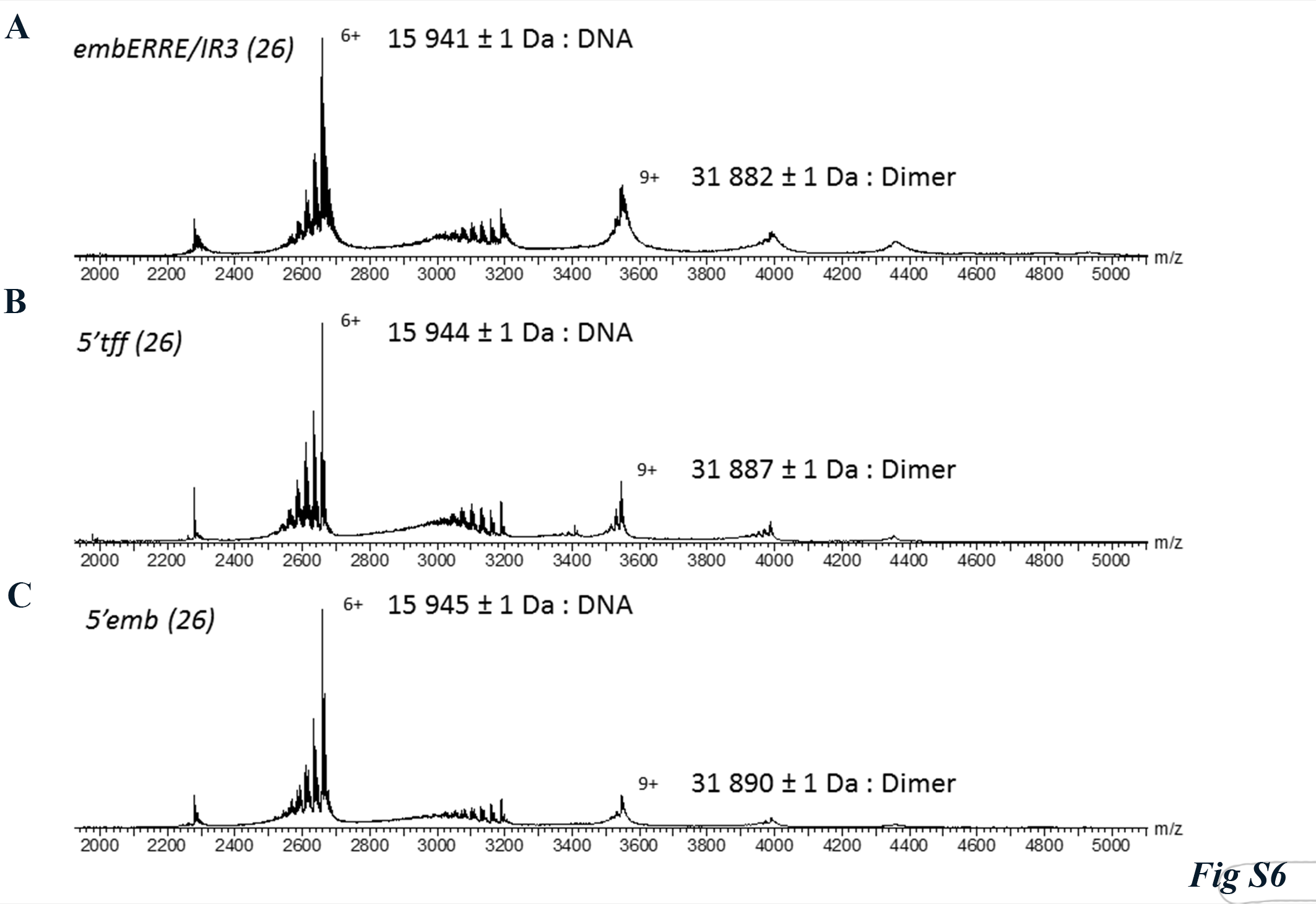

Supplement: Figure S6 — Non-denaturing Mass Spectrometry analysis of free DNA. Non-denaturing mass spectrometry analysis of free DNA complexes for (A) embERRE/IR3, (B) 5′tff, and (C) 5′emb, all being 26 bps long, recorded in the same non-denaturing conditions as the protein-DNA complexes are measured (Pi = 6 mbar; Vc = 140V). The charged states are indicated above the peaks. A small fraction of DNA dimer is observed under these experimental conditions. [file Image_6.TIF]

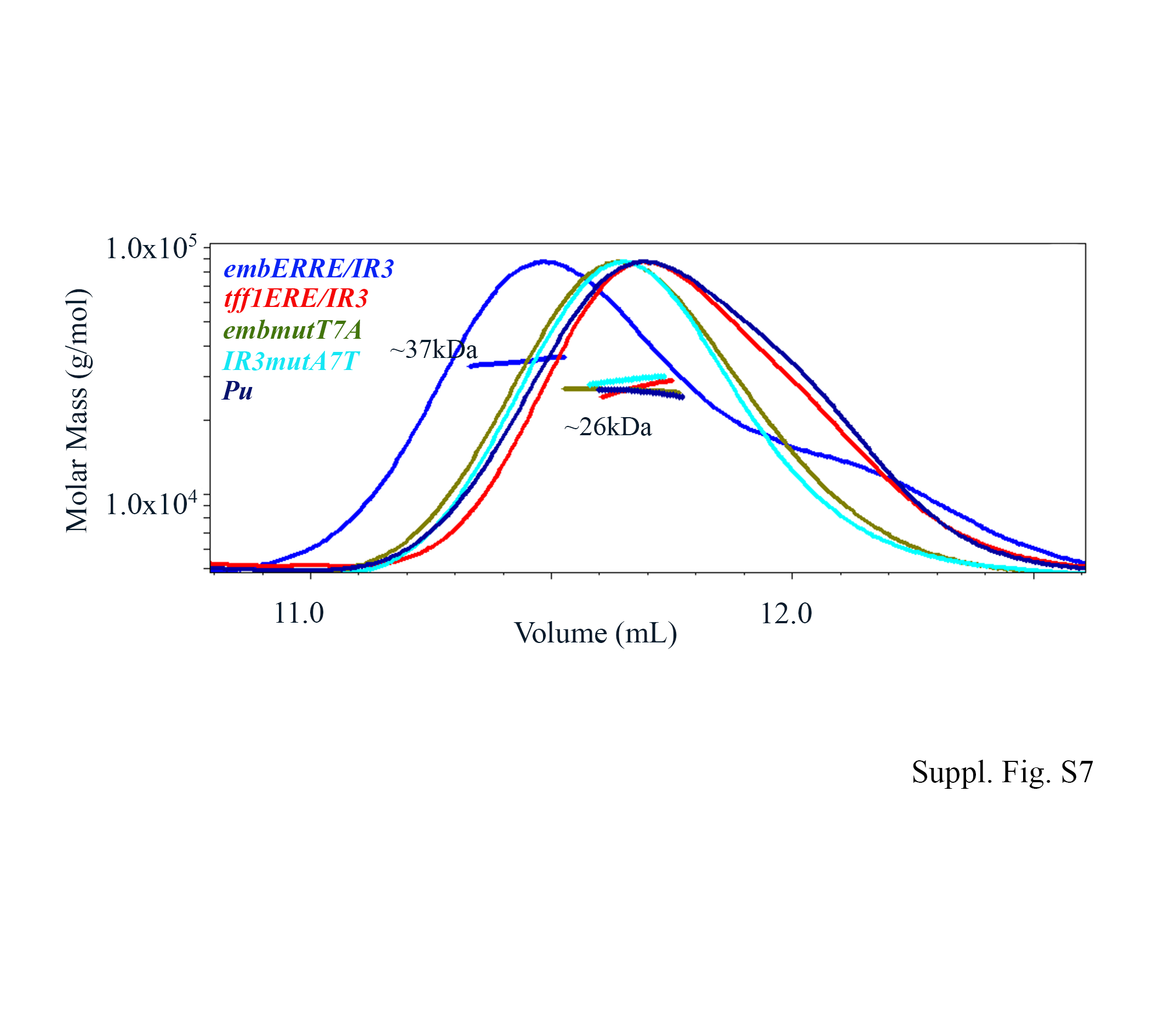

Supplement: Figure S7 — Size-exclusion chromatography-coupled multi-angle laser light scattering of ERRα DNA-binding domain (DBD)-DNA complexes. SEC-MALLS analysis of ERRα DBD bound to 26 bp embERRE/IR3 (blue), tff1 ERE/IR3 (red), embmutT7A (green), IR3mutA7T (cyan), and Pu (dark blue) (160 µM), showing the elution profile on a SEC S75 10/300 with the direct molar mass measurement of each elution peak. The elution profiles of the complexes with the mutant DNA complexes are eluted slightly earlier as tff1 ERE/IR3-ERRα DBD and the fraction of complex collected at the peak maximum is seen to contain a small fraction of dimeric DBD on DNA (see Figure 6D). [file Image_7.TIF]

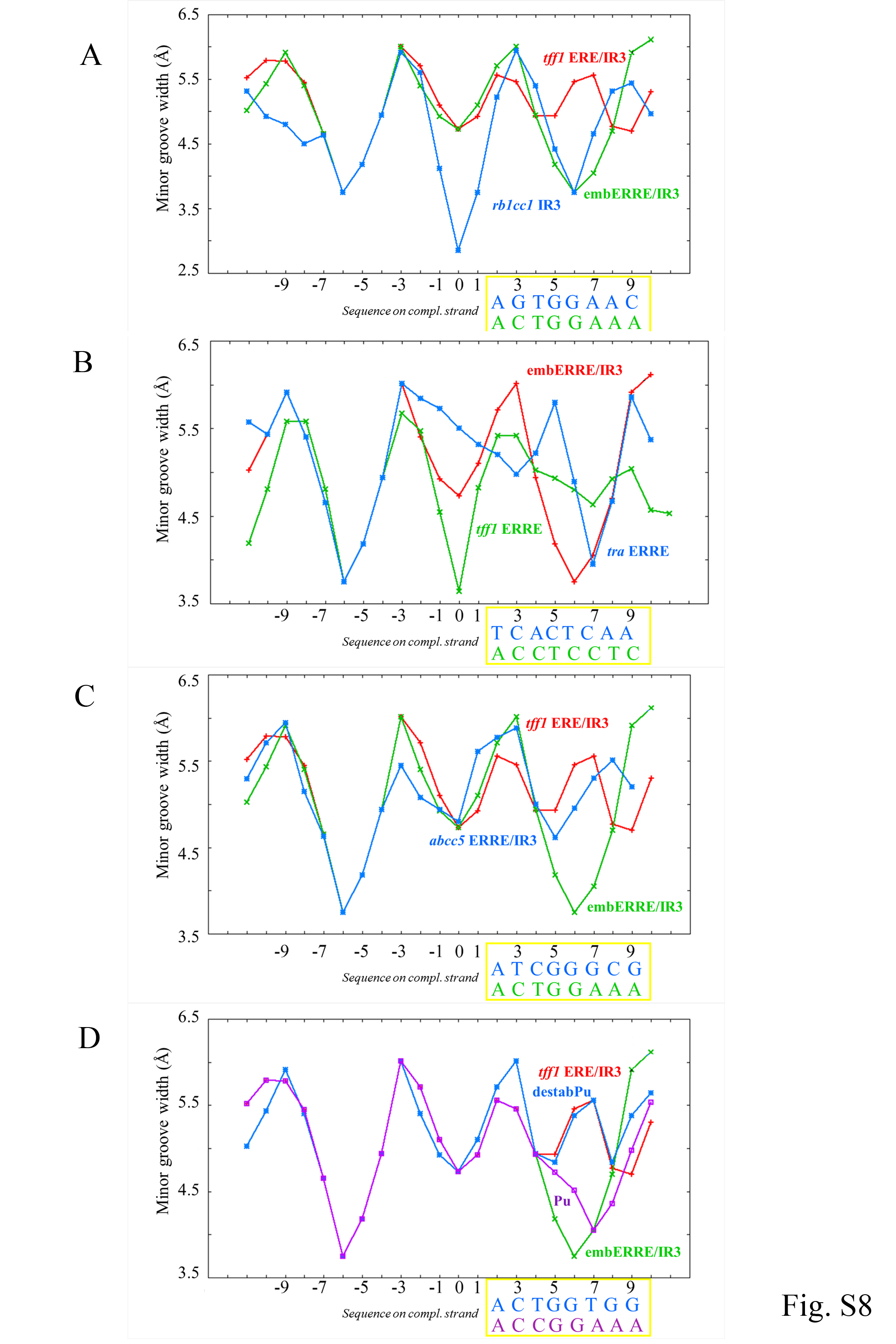

Supplement: Figure S8 — DNAshape predictions of other DNA fragments. Minor groove width (Å) is plotted as a function of base sequence for (A) rb1cc1 IR3 (blue) and compared to those of embERRE/IR3 (green) and tff1 ERE/IR3 (red). The position of the bases is given with respect to the central bp of the spacer (position 0). The sequences of the complementary strand of rb1cc1 IR3 (blue) and embERRE/IR3 (green) are given for positions (+2) to (+9) and comprise the second half-site and the two first flanking nucleotides; (B) tra estrogen-related response element (ERRE) (blue) and tff1 ERRE (green) compared to those of embERRE/IR3 (red). The position of the bases is given with respect to the central bp of the spacer (position 0). The sequences of the complementary strand of tra ERRE (blue) and tff1 ERRE (green) are given for positions (+2) to (+9) and comprise the second half-site and the two first flanking nucleotides; (C) abcc5 ERRE/IR3 (blue) and compared to those of embERRE/IR3 (green) and tff1 ERE/IR3 (red). The position of the bases is given with respect to the central bp of the spacer (position 0). The sequences of the complementary strand of abcc5 ERRE/IR3 (blue) and embERRE/IR3 (green) are given for positions (+2) to (+9) and comprise the second half-site and the two first flanking nucleotides; (D) the mutants destabPu (blue) and Pu (purple) and compared to those of embERRE/IR3 (green) and tff1 ERE/IR3 (red). The position of the bases is given with respect to the central bp of the spacer (position 0). The sequences of the complementary strand of destabPu (blue) and Pu (purple) are given for positions (+2) to (+9) and comprise the second half-site and the two first flanking nucleotides. [file Image_8.TIF]
